# Rethinking the immune properties of bilirubin in viral hepatitis: from bench to bedside

**DOI:** 10.1038/cti.2015.37

**Published:** 2015-12-11

**Authors:** Karla F Corral-Jara, Jorge L Trujillo-Ochoa, Mauricio Realpe, Arturo Panduro, Sonia Roman, Nora A Fierro

**Affiliations:** 1 Unidad de Inmunovirología, Servicio de Biología Molecular en Medicina, Hospital Civil de Guadalajara ‘Fray Antonio Alcalde', Guadalajara, Mexico; 2 Departamento de Biología Molecular, Centro Universitario de Ciencias de la Salud, Universidad de Guadalajara, Guadalajara, Mexico; 3 Departamento de Fisiologia, Centro Universitario de Ciencias de la Salud, Universidad de Guadalajara, Guadalajara, Mexico; 4 Departamento de Medicina Veterinaria, Centro Universitario de Ciencias Biológicas y Agropecuarias, Universidad de Guadalajara, Guadalajara, Mexico; 5 Servicio de Biología Molecular en Medicina, Hospital Civil of Guadalajara ‘Fray Antonio Alcalde', Guadalajara, Mexico; 6 Departamento de Clínicas Médicas, Centro Universitario de Ciencias de la Salud, Universidad de Guadalajara, Guadalajara, Mexico

## Abstract

Communication between the immune system and metabolic components can be exemplified by the process of heme catabolism. The immunomodulatory functions of the enzymes, substrates and active products related to catabolism of the heme group have been extensively studied. Bilirubin (BR), the final breakdown product of heme, is primarily considered to be a toxic waste product but has recently been considered to be an immunomodulatory metabolite. Through mechanisms that include intracellular signaling and transcriptional control, BR affects those immune cell functions that regulate cell proliferation, differentiation and apoptosis. During the pathogenesis of viral hepatitis, the heme degradation pathway is disrupted, resulting in changes to normal BR concentrations. These alterations have been previously studied mainly as a consequence of the infection. However, little is known about the potential immunomodulatory role played by BR in the development of infectious hepatocellular diseases. Differences in BR levels in the context of viral hepatitis are likely to provide important insights into the metabolite-mediated mechanisms controlling the immune responses underlying both the long-term persistence of hepatitis C virus (HCV) infection and the resolution of hepatitis A virus (HAV) infection during the acute phase. In this review, the cross-talk between heme catabolism and immune function is described in detail. Special emphasis is given to discoveries that hold promise for identifying immunologic features of metabolic products in the resolution of viral diseases.

Throughout history, scientists have been interested in the yellow color imparted to the sclera and skin in disease-specific circumstances. However, it was only 100 years ago that the biochemical process that results in the elevation of bilirubin (BR) in the plasma and whose main clinical characteristic is jaundice was quantified by a diagnostic test.^1^ Since then, research efforts have been conducted to elucidate the biochemical and physiological characteristics of BR. Until 30 years ago, the breakdown of heme to BR was simply considered to be one step of many in the elimination of senescent heme.^2^ Today, it is understood that the products of this reaction actually provide important regulatory functions as second messengers, scavengers and antioxidants. Moreover, anti-inflammatory and immunomodulatory effects have been associated with this process.^3^

Heme is released from hemoglobin, myoglobin and cytochrome proteins under normal circumstances. Heme oxygenase-1 (HO-1), whose activity is induced by pro-oxidative stimuli, degrades heme to generate three active products: carbon monoxide, ferrous iron (Fe^2+^) and biliverdin (BV). BV is rapidly converted into BR by biliverdin reductase (BVR). In the liver, BR is metabolically conjugated with glucuronic acid by the enzyme glucuronyltransferase, with the resulting conjugated BR (CBR) being soluble in water and, therefore, able to be excreted with urine and feces (Figure 1).^3^ In humans, when heme degradation occurs under normal conditions, CBR is rapidly excreted into bile and removed from the body through the gut. Thus, the amount of CBR present in the serum of healthy subjects is minimal (<10% of measured total BR), and CBR values are maintained below 0.3 mg dl^−1^.^2^ Approximately 250 mg day^−1^ of BR is produced by healthy human adults; 75% of this is derived from circulating hemoglobin, with the remaining 25% derived from the catabolism of other heme compounds, primarily cytochromes.^2^ Elevated levels of BR in the serum are associated with liver disease. In fact, BR concentration in the serum is an important component in the calculation of the Child-Pugh, FibroTest and the MELD (Model for End-Stage Liver Disease) discriminant scores that are important for estimating the prognosis of patients with liver disease.

Infections with hepatotropic viruses result in the elevation of serum aminotransferase activity and the elevation of serum-associated BR. Hepatitis A virus (HAV) is a hepatotropic virus that induces an acute infection resulting in a wide range of clinical conditions leading to the final elimination of the virus by a specific and effective host immune response. HAV infections are not associated with chronic liver disease.^4^ During HAV infection, heme degradation is interrupted in the final stages, leading to the deregulation of internalization and excretion of BR by hepatocytes that results in increased CBR values (>0.3 to 6 mg dl^−1^).^2^ In contrast, infection with another hepatotropic agent, hepatitis C virus (HCV), which is usually asymptomatic in the acute phase, leads, most often, to a lifelong chronic infection. However, it is known that a small subset of patients are capable of clearing HCV during the acute phase of infection.^5^ A common characteristic of chronic HCV infection is the episodic elevation of serum aminotransferase levels, whereas BR levels do not exceed 1 mg dl^−1^ in these patients.^6^

The exact mechanisms that contribute to the resolution of HAV and HCV infections in the acute phase have not been determined. However, it is accepted that the progression of these diseases is intrinsically restricted by host immunity and may also be affected by host-metabolic components.^4^ Given the immunomodulatory roles proposed for BR, it is plausible that changes in the levels of this metabolite may constitute a mechanism that contributes to differential outcomes in the liver diseases caused by hepatotropic viruses.

## HEME METABOLISM AND IMMUNE MODULATION

How BR can regulate the immune system, particularly those events related to the progression of viral hepatitis, is not well understood. However, enzymes and products involved in the degradation of heme, which is obtained from hemoglobin, to BR have previously been connected to immunological processes.^3^

### HO-1 and anti-inflammatory immune response against viruses

Macrophages are the main cells responsible for the degradation of heme. The first catabolic reaction involves HO-1 activity that gives rise to BV and, consequently, to BR; therefore, an increase of this enzyme elevates both metabolites. The direct link between HO-1 and inflammation was shown initially in an HO-1 knockout mouse model. These mice were highly vulnerable to experimentally induced inflammation mediated by treatment with the classical proinflammatory mediator endotoxin.^7^ In another study, the lymph nodes of HO-1-deficient mice demonstrated a relative deficiency of CD3- and B220-positive cells, significantly higher baseline serum IgM levels and higher levels of proinflammatory T helper type 1 (Th1) cytokines compared with wild-type mice.^8^ These findings suggest that the absence of HO-1 results in a Th-1-weighted shift in the cytokine response and that HO-1 may be essential in the modulation of inflammatory processes, particularly those related to viral diseases.

HO-1 is constitutively expressed in human CD4^+^ CD25^+^ T regulatory (Treg) cells.^3^ This expression pattern may be essential for the anti-inflammatory role of the enzyme. However, analyses of the crosstalk between HO-1 and Foxp3 (forkhead box P3)-related T regulatory function remain controversial. It has been shown that transfection of Foxp3 into Jurkat T cells induces the expression of HO-1. The same studies revealed that Foxp3 and HO-1 have the same expression pattern in T cells and suggested that the suppressive functions of peripheral CD4^+^CD25^+^Foxp3^+^ Treg cells are associated with an increased expression of HO-1.^9^ However, it has also been reported that HO-1 induction by chemical components does not result in Foxp3 expression in CD4^+^CD25^−^ T cells, suggesting that HO-1 activity is not essential for the Treg phenotype.^10^ Moreover, the induction of experimental autoimmune encephalomyelitis in mice with a myeloid-specific HO-1 deficiency leads to the development of an exacerbated and nonremitting clinical disease that correlates with an enhanced infiltration of Th17 cells and persistent activation of antigen-presenting cells.^11^ Together, these findings suggest that during pathological circumstances, Th17 and Treg phenotypes and activities may be related to HO-1 function.

Infection with Sendai virus in a model of macrophage-specific HO-1 deficiency resulted in reduced production of interferon-β (IFN-β) and impaired phosphorylation of interferon regulatory factor-3, suggesting that HO-1 plays a key role in triggering antiviral responses.^11^ This is consistent with observations that pharmacologically induced expression of HO-1 with hemin led to reduced hepatitis B virus (HBV) replication *in vitro* in HBV-infected HepG2.2.15 hepatoma cells^12^ as well as the observed reduction in levels of HBV core protein in a mouse model for acute hepatitis following the induction of HO-1 by cobalt-protoporphyrin-IX treatment.^13^ The potential antiviral effects of HO-1 during viral hepatitis are consistent with its association to liver disease. Functional polymorphic dinucleotide guanosine/thymine repeat regions are present in the proximal promoter region of HO-1, encompassing nucleotides −284 to −198, relative to the transcription start repeat regions in the HO-1 promoter. The reported number of guanosine/thymine repeats ranges from 12 to 40, although a bimodal distribution exists in most populations with the main alleles being composed of 24 or 30 repeats.^14^ The proportion of short allele (<22 (guanosine/thymine) repeats) frequency in the HO-1 promoter appears to be a risk factor for developing neonatal hyperbilirubinemia.^15, 16^

In agreement with the putative role of HO-1 in viral infection, *in vitro* hemin induction of HO-1 results in the suppression of HIV replication in previously inoculated cells, whereas in a humanized mouse model of HIV pathogenesis, HO-1 induction suppressed infection at a clinically relevant dose.^17^ Thus, induction of HO-1 may represent a novel antiviral strategy. This is supported by the fact that the HIV protease inhibitor ritonavir has been suggested to function, in part, by inducing HO-1.^18^ However, inhibition of HO-1 in cytomegalovirus-pp65-pulsed peripheral mononuclear cells resulted in increased antiviral T-cell activation and the generation of a higher proportion of memory T cells (CD45RA^−^CD62L^−^) capable of secreting IFN-γ and granzyme B.^19^ Thus, these findings suggest that the effect of HO-1 on viral infection is dependent on the particular viral etiology.

In humans, HO-1 deficiency is associated with susceptibility to oxidative stress and an increased proinflammatory state that correlates with severe endothelial damage. To date, there have been only two human cases of HO-1 deficiency described,^20^ supporting the notion that HO-1 expression is indispensable to human physiology. In addition, as expected from its major role in the catabolism of the heme group, HO-1 importantly influences the levels of BV and BR and, therefore, may play a key role in the pathogenesis of liver disease, particularly that related to viral infections.

### BVR, an antioxidant and modulator of intracellular signaling pathways

BVR is expressed mainly in macrophages, the spleen and the liver and has been observed to be overexpressed in renal carcinoma cells and in the hippocampus of Alzheimer's patients.^21, 22^ This pattern underscores the pleiotropic features of BVR that include acting as a transcription factor in response to stress, modulating signal transduction based on its Ser/Thr/Tyr kinase activity, directing protein–protein interactions and activating signaling pathways.^21^

BVR gene expression is induced by several factors, including lipopolysaccharides, free radicals and stress.^21^ It is also upregulated by hypoxia and negatively regulated by nuclear factor-κB that binds to the BVR promoter element in a region 125 nucleotides upstream of the transcription start site.^23^ The role of BVR as a regulator of the redox cycle is widely known; *in vitro* studies with human diploid young fibroblasts stimulated with hydrogen peroxide show increased activity of BVR that confers protection to the cells.^21^ Conversely, it has been shown *in vitro* that overexpression of human BVR (hBVR) in HeLa cells has a limited role in protection against hydrogen peroxide-induced damage.^24^ These data show that the modulatory role of the antioxidant BVR depends largely on the microenvironment and the cell type.

The hBVR has a transmembrane region. The enzymatic conversion of BV to BR on the surface of macrophages by hBVR initiates a signaling cascade through tyrosine phosphorylation of the hBVR cytoplasmic tail. Phosphorylated hBVR then binds to the p85 subunit of phosphatidylinositol 3-kinase and activates the downstream signaling of Akt, increasing the production of interleukin (IL)-10.^25^ Interestingly, IL-10 is known to reduce inflammation and increased levels of IL-10 in the peripheral blood are found during chronic viral diseases, including HBV, HCV and HIV infections.^26^ Thus, changes in BVR activity may be related to the differential outcomes observed during these infections.

BVR participates in multiple signaling pathways, including insulin signaling in which BVR can be phosphorylated at Y228 by insulin receptor tyrosine kinase.^21^ In turn, BVR can phosphorylate insuline receptor substrate-1 (serine), the first substrate of the insulin receptor/insulin-like growth factor 1 receptor responsible for the adenosine triphosphate/adenine binding site, leading to blockage of the insulin pathway.^27^ In addition, an *in vitro* study using the 293 cell line revealed that hBVR forms a ternary complex with the extracellular signal-regulated kinases 1/2 (ERK1/2) and mitogen-activated protein kinase-1 and activates ERK/EST domain-containing protein 1 (Elk1) signaling regardless of insuline-like growth factor-1.^27^ Moreover, hBVR can join the activator protein-1 and -2 sites and regulate the direct transcription of activating transcription factor (ATF)/CREB, Elk, c-jun and c-fos. hBVR is also involved in the activity of protein kinase C-β, γ and ζ.^23^ Together, these data confirm the ability of BVR to modulate many different cellular functions.

During inflammation, nitric oxide plays an important role in pathogen clearance after phagocytosis. Using the endothelial isoform of nitric oxide synthase knockout (eNOS^−/−^) mouse model, the functional importance of BVR-eNOS was tested *in vivo* during acute hepatitis induced by TNF-α and D-galactosamine. In this model, a BVR-related protection against hepatitis was observed. This protection required BVR activity and resulted in the reduction of Toll-like receptor-4 (TLR4) expression and inhibition of its transcription, leading to reduced TNF-α levels.^25^ This strongly suggests that during acute hepatitis, BVR may interfere with the innate immune response and thus modulate immune function. This understanding could be used to improve future therapies for the treatment of infectious liver disease.

### Immune role of BR

Given that BR is a product of HO-1 enzyme activity, its effect on immune cell function was assumed to be similar to that of HO-1. However, a variety of experiments support a direct role of BR in immune control. The *in vitro* incubation of immune cells with unconjugated BR at clinically relevant concentrations resulted in an inhibition of B-cell proliferation, the production of cytokines related to macrophage function and the induction of both the extrinsic and intrinsic apoptotic signaling pathways.^28^ Consistent with this, *in vitro* data show that CBR is able to induce IL-6 and TNF-α secretion in human peripheral blood lymphoid cells in a dose-dependent manner.^29^ Interestingly, this effect is enhanced during HAV infection.^30, 31^

Some molecular mechanisms have been already proposed for the ability of BR to inhibit cell growth: downregulation of mitogen-activated protein kinase signaling pathways and the prevention of nuclear translocation of nuclear factor-κB are two possibilities.^32, 33, 34^ It has also been demonstrated that BR inhibits protein kinase C and Ca^2+^-calmodulin-dependent protein kinases at concentrations ranging from 20 to 125 μM and inhibits inducible signal transducer and activator of transcription 1 (STAT-1) and IκB phosphorylation.^32^ Moreover, BR has been reported to directly block nuclear factor-κB binding to DNA.^35^ *In vivo*, intraperitoneal administration of BR influences the expression of Fc receptors, and discrete changes in BR concentration result in upregulation of costimulatory receptors in immune cells.^32, 36^ These observations are consistent with suggestions that BR might regulate immune functions, particularly those associated with membrane receptors, because of its high lipophilic chemical nature.^36^ In addition, these findings suggest that BR is able to control signaling pathways and influence immune functions at several levels.

Control of apoptosis is an important component of disease progression, and it is physiologically relevant during oxidative stress. In rat hepatocytes, it was observed that the antioxidant properties of both conjugated and unconjugated BR inhibited the induced accumulation of bile acids in a dose-dependent manner, suppressing the generation of reactive oxygen species. This, in turn, prevented hepatocellular injury and suggests that hyperbilirubinemia may have a protective role in liver disease.^37^

Of note, the regulation of serum BR concentrations is a complex process involving multiple organs, enzymes and membrane transport systems.^2^ Thus, in terms of health and infectious viral diseases, discrete changes in BR levels may result in different outcomes. It is thought that the antiviral functions related to the heme metabolic pathway are not exclusive to viral hepatitis; heme, BV and BR can inhibit HIV protease despite the fact that these viral proteases differ markedly in primary structure and reaction mechanisms.^38^ Thus, the antiviral effect of BR may be generalized to other viral infections.

## HEME METABOLISM AND MODULATION OF TYPE A AND C VIRAL HEPATITIS

Although the concept of antiviral activity modulated by heme metabolites was suggested many years ago, the potential immune modulatory roles of BR and the different components involved in its biosynthetic pathway in type A and C viral hepatitis have not been clearly defined. Changes in hepatic enzymes, including aspartate aminotransferase and alanine aminotransferase, as well as changes in the concentration of BR, have been associated with liver injury during viral hepatitis. In particular, CBR values >2 mg dl^−1^ are linked with cholestasis, a condition in which substances normally excreted into bile are retained. These CBR levels are associated with jaundice, a major characteristic of acute liver injury, whereas CBR levels above 15 mg dl^−1^ are related to fulminant hepatitis.^39^ Therefore, it is plausible that the interplay between the BR levels and the immune response to HAV and HCV influence the severity of disease.

### HAV infection: the virus, transmission and clinical outcomes

HAV is a positive-strand RNA picornavirus and is a prominent cause of acute hepatitis cases reported worldwide. Transmission of the virus occurs via the fecal–oral route through contaminated food or drinking water. Although improved hygiene and vaccination have reduced the overall HAV infection rate, the virus remains widespread in developing countries where the average age at infection has increased in recent years and results in a more severe hepatitis.^4^

The HAV-related clinical course is divided into four periods: (1) an incubation period characterized by active viral replication that has a duration between 15 and 50 days, depending on the initial inoculum; (2) the pre-jaundiced or prodromal period that exists in more than 75% of cases and shows nonspecific symptoms that can be attributed to other diseases until jaundice appears; this period lasts between 2 and 3 days; (3) the jaundiced period coincides with the onset of jaundice in some patients; and (4) the resolution period.^40^

### Immune response to HAV: the roles of BR

The humoral immune response to HAV is characterized by the specific production of IgM antibodies that target the VP1 viral protein and IgG antibodies specific for the VP1 and VP3 viral proteins.^41^ Although the early antibody response is composed largely of IgM, IgG may also be present shortly after the onset of symptoms and then persists for life to confer protection against reinfection.^4^ IgM antibodies are typically short lived during HAV infection, and their maximum levels coincide with a marked elevation in serum aminotransferase activity, viremia and maximum serum levels of BR, reaching CBR values above normal (>0.3 to 6 mg dl^−1^). Interestingly, a pronounced suppression of antibody levels against tetanus, pertussis and diphtheria in children with hyperbilirubinemia has been described, suggesting a role for BR in the humoral immune response.^35^

In contrast to the cellular responses raised against most viral infections in which CD8^+^ T cells play a predominant role, the resolution of HAV seems to rely on CD4^+^ T cells that act early in infection and produce numerous cytokines, including IFN-γ, TNF-α, IL-2 and IL-21.^42^ Our research group has reported that HAV-infected children show overexpression of TNF-α, IL-1, IL-6, IL-13 and monocyte chemoattractant protein-2 (MCP-2), all of which correlate with high serum levels of CBR (>2 mg dl^−1^). However, in patients with low CBR serum levels (>0.3 to 2 mg dl^−1^), cytokines associated with hepatitis-induced inflammation, such as TGF-β and IL-8, are predominantly expressed.^29, 31^ This strongly suggests that BR affects cytokine profile expression during HAV infection and may be able to influence the severity of disease.

Interestingly, a transient inhibition of Treg cellular function has been described during HAV viremia, and Treg cell reactivation occurs once HAV is cleared, allowing for the adequate control of inflammation.^43^ The exact mechanism by which this inhibition occurs in Treg cells is not yet clear. It has been proposed that a direct interaction between HAV and its receptor on the T-cell surface (HAVCR1/TIM-1) transitorily inhibits T regulatory function during viremia.^43^ Although the pathway responsible for HAVCR1/TIM-1 modulation has not been clearly defined, it has been reported that discrete levels of unconjugated BR suppress CD4^+^ T cell reactivity by mechanisms that include prevention of CD28, B7-1 and B7-2 up-regulation, resulting in a reduction of costimulatory signals.^36^ Thus, changes in BR levels during infection may affect the expression of co-receptors, including HAVCR1/TIM-1, on the cellular surface, and this might modulate Treg function. In addition, patients with cholestatic chronic jaundice demonstrate associated T-cell proliferation impairments and increased incidence of other infections.^44^

**Figure 2:**
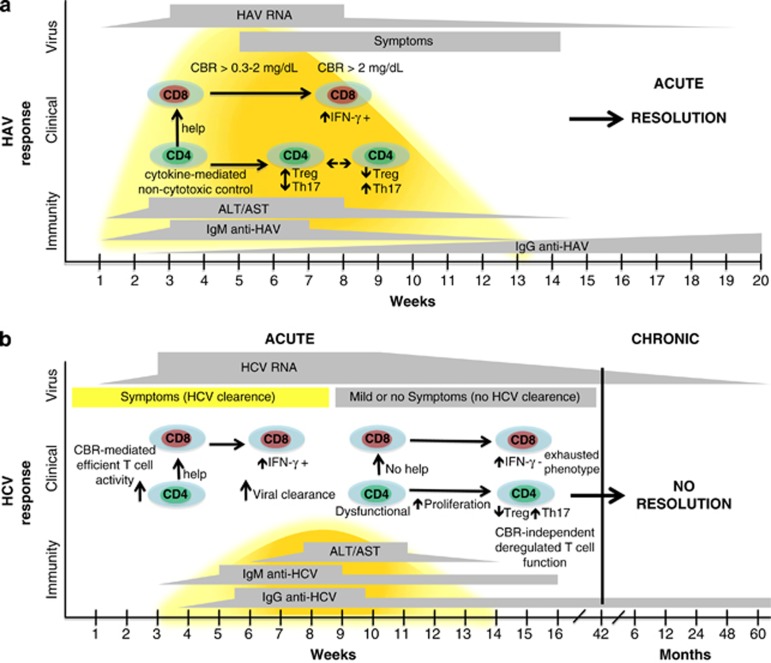
Immunologic, clinical and virologic course of HAV and HCV infections. (**a**) The immune response to HAV infection relies on CD4^+^ T cells that act during the first steps of infection and produce numerous cytokines. A transitory inhibition of T regulatory function is observed during viremia and reactivation of T regulatory activity occurs once HAV has been cleared. This mechanism may be related to changes in BR levels during infection (see text). (**b**) A strong and sustained CD4^+^ T-cell response is critical for the clearance of acute HCV infection. In the absence of an effective CD4^+^ T-cell response, CD8^+^ T cells develop an exhausted phenotype that allows for disease progression. This outcome may be related to the fact that a minority of patients develop a substantial elevation in their BR levels, leading to overt jaundice (see text).

Supporting the putative modulation of cellular immune responses during HAV infection, CBR levels >0.3 to <2 mg dl^−1^ result in the secretion of TGF-β and increased STAT-1 and STAT-5 phosphorylation, whereas CBR levels >2 mg dl^−1^ correlate with a reduction in the proportion of peripheral blood lymphoid cells positive for STAT-5 phosphorylation and increased levels of IL-6.^29^ Interestingly, it is known that STAT-5 acts as an important mediator of Foxp3 expression and Treg function and that the release of TGF-β in the serum during acute viral infection inhibits antigen-specific T-cell activation and proliferation as a result of modulating Treg cell activity.^45^ Thus, it is tempting to speculate that the global immune response against HAV may be influenced by the CBR levels in serum. During HAV infection, low levels of CBR (>0.3–2 mg dl^−1^) may result in Treg cell activation, whereas high levels of CBR (>2 mg dl^−1^) could induce Treg cell inactivation. Together, these events may result in efficient control of the inflammatory process that later will lead to the resolution of acute viral infection (Figure 2a). This hypothesis is supported by studies describing the induction of tolerance after administration of BR to transplant recipients, resulting from *de novo* generation of Treg cells in a murine cardiac allograft model and a murine model of pancreatic islet transplant.^46, 47^ Moreover, the ability of BR to inhibit T-cell proliferation and its ability to decrease IL-2 production in human lymphocytes has previously been described.^3^

Conversely, it has been reported that high levels of BR could induce apoptosis in reactive CD4^+^ T cells, and *in vitro* models have shown that BR concentrations >25 mM induce apoptosis in both CD4^+^ T cells and neutrophils.^32^ In support of this, BR has been observed to suppress inflammation and increase antioxidant enzyme generation in activated neonatal neutrophils by downregulating the lipopolysaccharide-induced generation of IL-8.^48^ It is interesting that a reduction in IL-8 secretion is found in HAV-infected patients with CBR levels >2 mg dl^−1^. Given that clinically relevant concentrations of BR can induce apoptosis in neutrophils and that these cells are a primary source of IL-8, it is plausible that the changes in the proportion of neutrophils because of high concentrations of BR may result in IL-8 downregulation.^29^ Hence, discrete CBR values may differentially alter cellular functions during HAV infection, specifically those related to neutrophils and CD4^+^ T cells.

HAV infection has been extensively associated with protection against the development of autoimmune diseases and allergies, as is observed in nonindustrialized countries where high incidence of HAV coincides with a reduced proportion of allergies.^49^ However, the exact mechanisms involved in this process have not been determined. Recent findings suggest that during HAV-related clinical courses, increased levels of IL-17F may play a protective role against allergies by reducing IgE levels, and this is supported by the trend toward IgE level reduction in HAV-infected patients with a CBR >2 mg dl^−1^ who also show the highest levels of IL-17F.^30^ Thus, discrete CBR levels resulting from infection may drive anti-inflammatory immune responses that, in turn, may protect against allergens or autoimmune mediators. In addition, during autoimmune processes, *in silico* models suggest that BR may bind to the antigenic peptide-binding groove of human leukocyte antigen molecules resulting in the blockade of antigenic peptide presentation to T-cell receptors, leading to eventual suppression of the immune response.^50^ This model is consistent with the fact that BR treatment results in a downregulation of inducible MHC class II expression that has a notable effect on Ag presentation to CD4^+^ T cells.^32^ Thus, under most pathological conditions, including HAV infection, changes in normal BR concentration may modulate specific immune responses and therefore result in differential disease outcomes.

### HCV virus: transmission and clinical outcomes

HCV infection is caused by a positive-strand RNA flavivirus. Transmission primarily occurs via parenteral routes and particularly by unsafe injections. Infection is asymptomatic in most (70–80%) cases, whereas the remaining patients (20–30%) develop clinical symptoms. Some infected individuals can resolve their infections (15%), whereas most develop chronic hepatitis (85%) and rarely, fulminant hepatitis. In cases of chronic hepatitis, 80% maintain the disease, whereas 20% go on to develop cirrhosis and hepatocellular carcinoma.^51^

The incubation period for HCV ranges from 2 to 26 weeks, with a mean of between 6 and 12 weeks. HCV RNA is detectable in the blood for 1 to 3 weeks post infection, coincident with elevations in serum transaminases and the presentation of symptoms. In symptomatic acute HCV infection, anti-HCV antibodies are detected in only 50 to 70% of patients; in the remaining patients, the anti-HCV antibodies emerge after 3 to 6 weeks. During chronic infection, circulating HCV RNA persists in many patients, including >90% of patients with chronic disease, despite the presence of neutralizing antibodies.^41^ During acute infection, serum aminotransferases often reach values below 1000 IU ml^−1^ and may return to normal levels. In the majority of cases, acutely infected patients develop mild constitutional symptoms, and only a minority of patients develop sufficient elevations in BR to lead to overt jaundice.^51^

### Heme metabolism and immune response to HCV

The significant role played by heme-related enzymes and metabolites in viral hepatitis is supported by studies demonstrating that BV inhibits the viral protease, an enzyme critical for the pathogenesis of HCV. Decreased levels of the HCV NS3/4A protease correlate with lower viral replication in *in vitro* infected hepatocytes because of triggering of the antiviral interferon response.^52, 53^ In addition, BVR upregulation has been observed in chronically HCV-infected patients with a sustained response to treatment relative to nonresponding patients.^45^ Furthermore, the induction of HO-1 in HCV infection has been shown to decrease viral replication, as well as protect against oxidative damage.^54^ Taken together, changes in the activity or levels of heme-related enzymes and metabolites may be related to a protective role against hepatocellular injury during HCV-mediated viral hepatitis and could represent potential targets for novel antiviral approaches based on this metabolic pathway.

It is commonly accepted that a strong and sustained T-cell response is critical for a positive outcome in HCV infection. Both CD4^+^ and CD8^+^ T cells have been shown to play major roles during this process. In particular, the production of IFN-γ by Th1 cells is required for an adequate immune response against HCV.^41^ The mechanisms behind the antiviral properties of HO-1, BV and BR have not been clearly defined. However, the recognized ability of BR to modulate T-cell proliferation may explain the weak and functionally impaired CD4^+^ T cell responses reported in patients who fail to clear HCV.^3, 41^ In addition, it is plausible that, as suggested for HAV infection, discrete levels of CBR may result in Treg cell inactivation, promoting productive T-cell function. This, in turn, would result in a release of adequate levels of IFN by CD8^+^ T cells to drive subsequent antiviral activity in those individuals who spontaneously clear the HCV during the acute phase of infection (Figure 2b). This model is consistent with retrospective studies of HCV-infected patients who revealed an association between the presence of symptoms and jaundice during the acute stage and spontaneous viral clearance.^51, 55^ Based on these data, it is possible that those mild acute HCV infections that are not associated with a discernable increase of serum CBR cause the host to progress to chronic disease. In that case, BR may play an important role in the resolution of HCV infection during the acute stage.

The finding that, during HCV infection, the absence of an effective CD4^+^ T-cell response results in the development of an exhausted CD8^+^ T-cell pool has been attributed to chronic antigen-specific stimulation.^41^ Considering that discrete CBR levels may be necessary to modulate T-cell function through Treg activity during mild acute infections, suboptimal levels of CBR may contribute to the exhausted T-cell response, allowing for viral adaptation to the host immune response and concomitant development of chronic infection. In this regard, mild and advanced HCV-related fibrosis is associated with CBR levels between 0.28 and 0.48 mg dl^−1^, respectively.^2, 56^ An inverse relationship between unconjugated BR levels and the advanced liver fibrosis caused by HCV has been reported,^56^ therefore suggesting that during the development of chronic HCV infection, discrete changes in BR levels may be beneficial for controlling liver injury.

The roles of Treg cells in HCV infection could span from the suppression of anti-HCV T-cell responses to the downregulation of whole immune responses in those cases where it would cause liver damage.^9^ There are clinical data revealing an increase of IL-17 levels in HCV-infected patients who also showed increased alanine transaminase values. Given that the Th17 cell subpopulation is well known for regulating inflammation in chronic hepatitis C infection, these cells might be associated with the development of liver injury.^14, 20^ These increasing lines of evidence support the notion that modulation of disease progression is based on a fine balance between the Treg and Th17 cellular phenotype and that the function of those subtypes could be affected by subtle changes in the levels of BR and heme-related metabolites. Further studies are needed to test the hypothetical role of BR in the context of viral hepatitis clinical outcomes.

## REMARKS

Viral hepatitis causes considerable morbidity and mortality worldwide. Strategies have been developed to specifically target HCV, given the chronic nature of the infection associated with this agent. However, a detailed understanding of the exact mechanisms responsible for spontaneous viral resolution of both HAV and HCV during the acute phase of infection has not yet been achieved. Based on the recognized immune functions of the heme group and its related metabolites, it is plausible that these molecules may modulate immune function and result in the resolution of acute viral disease.

**Figure 3:**
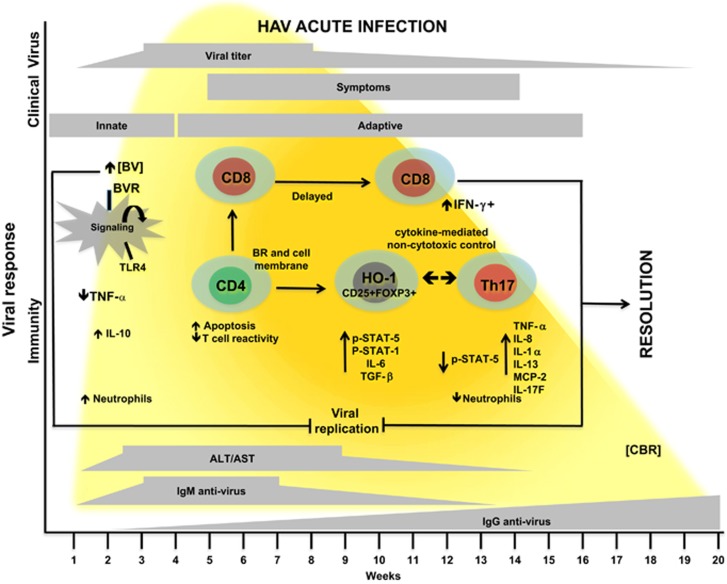
Proposed model of interactions that lead to favorable outcomes during acute HAV-associated liver disease: implication of heme-related metabolites. During the first weeks of HAV infection, the interaction between BV and BVR on the macrophage surface modifies intracellular signaling, resulting in downregulation of Toll-like receptor-4 (TLR4) and the subsequent reduction in TNF-α production. Low CBR levels result in increased STAT-5 phosphorylation, as well as IL-6 and TGF-β expression, suggesting that changes in either proportion of Treg cells or their function can control the inflammatory process. Augmented CBR levels during infection coincide with the initiation of the adaptive immune response. We propose that high CBR levels induce an overproduction of proinflammatory cytokines as a result of a reduction in STAT-5 phosphorylation. Because BR is highly lipophilic and directly interacts with cell membranes, BR-related immune functions may be associated with membrane receptors. Moreover, it is possible that the direct control of proteases mediated by HO-1, BVR and BR involves an effective induction of proinflammatory components. This coincides with a reduction in neutrophil numbers and IL-8 in the periphery. After viral clearance, these inflammatory components will be regulated by Treg cells, whose function could be related to HO-1 expression. In addition, BR has been associated with the induction of apoptosis in both T cells and neutrophils that may act as an additional mechanism to control the inflammatory environment.

Based on HAV infection, a model is proposed in which heme-related metabolites, including HO-1, BV, BVR and BR, mainly modulate CD4^+^ T-cell function leading to disease resolution (Figure 3). This model is in agreement with evidence supporting the role of CD4^+^ T cells in directly controlling HAV infection through the production of antiviral cytokines. Thus, noncytotoxic control of viral replication by CD4^+^ T cells could be a more general mechanism for resolving numerous viral infections regardless of CD8^+^ T-cell activity.

A better understanding of HAV-related immune pathogenesis, particularly those aspects related to metabolic components, could provide further insight into general mechanisms of immune evasion and control that may apply to other liver-tropic viruses, including HCV. The study of these metabolites may be beneficial in terms of designing novel therapeutic strategies to prevent the development of chronic liver diseases in the future. Moreover, considering the ability of the heme oxygenase system to inhibit multiple viral target sites from various viruses, further studies should focus on the evaluation of porphyrin-based antivirals *in vivo*, particularly in patients with multiple viral coinfections.

## Figures and Tables

**Figure 1:**
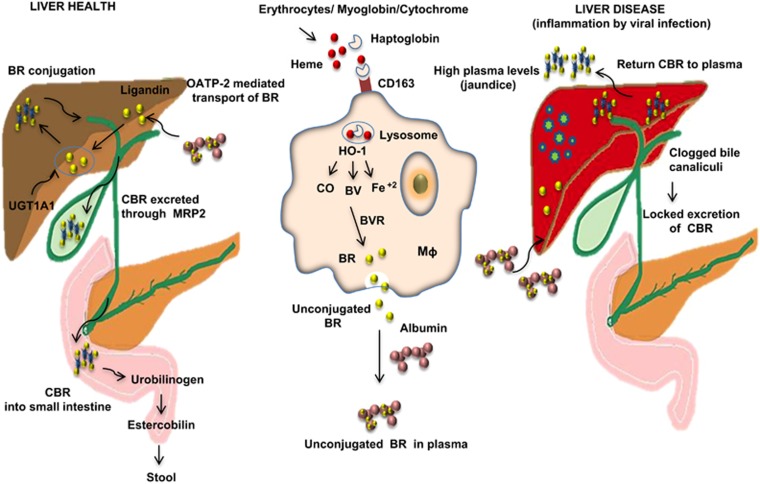
Major pathways involved in BR production, conjugation and excretion in healthy and liver disease conditions. HO-1 catalyzes the heme degradation reaction, yielding three biologically active products: carbon monoxide (CO), BV and Fe^2+^. BV is subsequently reduced to BR by BVR. Both the unconjugated BR and CBR forms are bound by albumin in the plasma and interstitial space. The OATP-2-mediated mechanism is responsible for transporting unconjugated BR into hepatocytes. BR is conjugated in the liver by the UGT family of enzymes, resulting in the water-soluble CBR capable of excretion with the urine and feces. Under healthy conditions, CBR is rapidly excreted into bile and removed from the body through the gut. Therefore, CBR concentrations in the serum of healthy subjects are minimal (CBR values are kept below 0.3 mg dl^−1^). Pathological conditions influence various pathways associated with BR excretion. During infection with hepatotropic viruses, hepatocytes prevent internalization and excretion of BR, resulting in serum CBR levels above normal (>0.3 mg dl^−1^). CD163, scavenger receptor; Mq, macrophage; MRP2, multidrug resistance-associated protein 2; OATP-2, organic anion-transporting polypeptide 2.

## References

[bib1] 1Papavramidou N, Fee E, Christopoulou-Aletra H. Jaundice in the hippocratic corpus. J Gastrointest Surg 2007; 11: 1728–1731. 17896166 10.1007/s11605-007-0281-1

[bib2] 2Levitt DG, Levitt MD. Quantitative assessment of the multiple processes responsible for bilirubin homeostasis in health and disease. Clin Exp Gastroenterol 2014; 7: 307–328. 25214800 10.2147/CEG.S64283 PMC4159128

[bib3] 3Jangi S, Otterbein L, Robson S. The molecular basis for the immunomodulatory activities of unconjugated bilirubin. Int J Biochem Cell Biol 2013; 45: 2843–2851. 24144577 10.1016/j.biocel.2013.09.014

[bib4] 4Walker CM, Feng Z, Lemon SM. Reassessing immune control of hepatitis A virus. Curr Opin Virol 2015; 11: 7–13. 25617494 10.1016/j.coviro.2015.01.003 PMC4456347

[bib5] 5Rehermann B, Nascimbeni M. Immunology of hepatitis B virus and hepatitis C virus infection. Nat Rev Immunol 2005; 5: 215–229. 15738952 10.1038/nri1573

[bib6] 6Ijaz B, Ahmad W, Javed FT, Gull S, Sarwar MT, Kausar H et al. Association of laboratory parameters with viral factors in patients with hepatitis C. Virol J 2011; 8: 361–369. 21777434 10.1186/1743-422X-8-361 PMC3154183

[bib7] 7Naito Y, Takagi T, Higashimura Y. Heme oxygenase-1 and anti-inflammatory M2 macrophages. Arch Biochem Biophys 2014; 564: 83–88. 25241054 10.1016/j.abb.2014.09.005

[bib8] 8Kapturczak MH, Wasserfall C, Brusko T, Campbell-Thompson M, Ellis TM, Atkinson MA et al. Heme oxygenase-1 modulates early inflammatory responses: evidence from the heme oxygenase-1-deficient mouse. Am J Pathol 2004; 165: 1045–1053. 15331427 10.1016/S0002-9440(10)63365-2 PMC1618611

[bib9] 9Choi BM, Pae HO, Jeong YR, Kim YM, Chung HT. Critical role of heme oxygenase-1 in Foxp3-mediated immune suppression. Biochem Biophys Res Commun 2005; 327: 1066–1071. 15652505 10.1016/j.bbrc.2004.12.106

[bib10] 10Biburger M, Theiner G, Schadle M, Schuler G, Tiegs G. Pivotal advance: heme oxygenase 1 expression by human CD4+ T cells is not sufficient for their development of immunoregulatory capacity. J Leukoc Biol 2010; 87: 193–202. 19797297 10.1189/jlb.0508280

[bib11] 11Tzima S, Victoratos P, Kranidioti K, Alexiou M, Kollias G. Myeloid heme oxygenase-1 regulates innate immunity and autoimmunity by modulating IFN-beta production. J Exp Med 2009; 206: 1167–1179. 19398754 10.1084/jem.20081582 PMC2715044

[bib12] 12Shen YM, Zhang HL, Wu YH, Yu XP, Hu HJ, Dai LH. Dynamic correlation between induction of the expression of heme oxygenase-1 and hepatitis B viral replication. Mol Med Rep 2015; 11: 4706–4712. 25633656 10.3892/mmr.2015.3278

[bib13] 13Protzer U, Seyfried S, Quasdorff M, Sass G, Svorcova M, Webb D et al. Antiviral activity and hepatoprotection by heme oxygenase-1 in hepatitis B virus infection. Gastroenterology 2007; 133: 1156–1165. 17919491 10.1053/j.gastro.2007.07.021

[bib14] 14Origassa CS, Camara NO. Cytoprotective role of heme oxygenase-1 and heme degradation derived end products in liver injury. World J Hepatol 2013; 5: 541–549. 24179613 10.4254/wjh.v5.i10.541 PMC3812456

[bib15] 15Katayama Y, Yokota T, Zhao H, Wong RJ, Stevenson DK, Taniguchi-Ikeda M et al. Association of HMOX1 gene promoter polymorphisms with hyperbilirubinemia in the early neonatal period. Pediatr Int 2015; 57: 645–649. 25625535 10.1111/ped.12591

[bib16] 16D'Silva S, Borse V, Colah RB, Ghosh K, Mukherjee MB. Association of (GT)n repeats promoter polymorphism of heme oxygenase-1 gene with serum bilirubin levels in healthy Indian adults. Genet Test Mol Biomarkers 2011; 15: 215–218. 21198350 10.1089/gtmb.2010.0132

[bib17] 17Devadas K, Dhawan S. Hemin activation ameliorates HIV-1 infection via heme oxygenase-1 induction. J Immunol 2006; 176: 4252–4257. 16547262 10.4049/jimmunol.176.7.4252

[bib18] 18Muhl H, Paulukat J, Hofler S, Hellmuth M, Franzen R, Pfeilschifter J. The HIV protease inhibitor ritonavir synergizes with butyrate for induction of apoptotic cell death and mediates expression of heme oxygenase-1 in DLD-1 colon carcinoma cells. Br J Pharmacol 2004; 143: 890–898. 15504750 10.1038/sj.bjp.0706023 PMC1575947

[bib19] 19Bunse CE, Fortmeier V, Tischer S, Zilian E, Figueiredo C, Witte T et al. Modulation of heme oxygenase-1 by metalloporphyrins increases anti-viral T cell responses. Clin Exp Immunol 2015; 179: 265–276. 25196646 10.1111/cei.12451 PMC4298404

[bib20] 20Grochot-Przeczek A, Dulak J, Jozkowicz A. Haem oxygenase-1: non-canonical roles in physiology and pathology. Clin Sci (Lond) 2012; 122: 93–103. 21992109 10.1042/CS20110147

[bib21] 21Florczyk UM, Jozkowicz A, Dulak J. Biliverdin reductase: new features of an old enzyme and its potential therapeutic significance. Pharmacol Rep 2008; 60: 38–48. 18276984 PMC5536200

[bib22] 22Barone E, Di Domenico F, Cenini G, Sultana R, Cini C, Preziosi P et al. Biliverdin reductase—a protein levels and activity in the brains of subjects with Alzheimer disease and mild cognitive impairment. Biochim Biophys Acta 2011; 1812: 480–487. 21241799 10.1016/j.bbadis.2011.01.005 PMC3042515

[bib23] 23Gibbs PE, Tudor C, Maines MD. Biliverdin reductase: more than a namesake - the reductase, its Peptide fragments, and biliverdin regulate activity of the three classes of protein kinase C. Front Pharmacol 2012; 3: 31–41. 22419908 10.3389/fphar.2012.00031 PMC3299957

[bib24] 24Maghzal GJ, Leck MC, Collinson E, Li C, Stocker R. Limited role for the bilirubin-biliverdin redox amplification cycle in the cellular antioxidant protection by biliverdin reductase. J Biol Chem 2009; 284: 29251–29259. 19690164 10.1074/jbc.M109.037119 PMC2785555

[bib25] 25Wegiel B, Otterbein LE. Go green: the anti-inflammatory effects of biliverdin reductase. Front Pharmacol 2012; 3: 47–55. 22438844 10.3389/fphar.2012.00047 PMC3306015

[bib26] 26Ni G, Wang T, Walton S, Zhu B, Chen S, Wu X et al. Manipulating IL-10 signalling blockade for better immunotherapy. Cell Immunol 2015; 293: 126–129. 25596475 10.1016/j.cellimm.2014.12.012

[bib27] 27Lerner-Marmarosh N, Miralem T, Gibbs PE, Maines MD. Human biliverdin reductase is an ERK activator; hBVR is an ERK nuclear transporter and is required for MAPK signaling. Proc Natl Acad Sci USA 2008; 105: 6870–6875. 18463290 10.1073/pnas.0800750105 PMC2383961

[bib28] 28Khan NM, Poduval TB. Immunomodulatory and immunotoxic effects of bilirubin: molecular mechanisms. J Leukoc Biol 2011; 90: 997–1015. 21807743 10.1189/jlb.0211070

[bib29] 29Castro-Garcia FP, Corral-Jara KF, Escobedo-Melendez G, Sandoval-Hernandez MA, Rosenstein Y, Roman S et al. Conjugated bilirubin affects cytokine profiles in hepatitis A virus infection by modulating function of signal transducer and activator of transcription factors. Immunology 2014; 143: 578–587. 24943111 10.1111/imm.12336 PMC4253506

[bib30] 30Trujillo-Ochoa JL, Corral-Jara KF, Escobedo-Melendez G, Realpe M, Panduro A, Roman S et al. T-helper 17-related cytokines and IgE antibodies during hepatitis A virus infection in children. Mem Inst Oswaldo Cruz 2015; 110: 263–266. 25946253 10.1590/0074-02760140309 PMC4489460

[bib31] 31Fierro NA, Escobedo-Melendez G, De Paz L, Realpe M, Roman S, Panduro A. Cytokine expression profiles associated with distinct clinical courses in hepatitis A virus-infected children. Pediatr Infect Dis J 2012; 31: 870–871. 22531242 10.1097/INF.0b013e318258e808

[bib32] 32Liu Y, Li P, Lu J, Xiong W, Oger J, Tetzlaff W et al. Bilirubin possesses powerful immunomodulatory activity and suppresses experimental autoimmune encephalomyelitis. J Immunol 2008; 181: 1887–1897. 18641326 10.4049/jimmunol.181.3.1887

[bib33] 33Fernandes A, Falcao AS, Silva RF, Brito MA, Brites D. MAPKs are key players in mediating cytokine release and cell death induced by unconjugated bilirubin in cultured rat cortical astrocytes. Eur J Neurosci 2007; 25: 1058–1068. 17331202 10.1111/j.1460-9568.2007.05340.x

[bib34] 34Ollinger R, Kogler P, Troppmair J, Hermann M, Wurm M, Drasche A et al. Bilirubin inhibits tumor cell growth via activation of ERK. Cell Cycle 2007; 6: 3078–3085. 18073533 10.4161/cc.6.24.5022

[bib35] 35Schwartz HP, Haberman BE, Ruddy RM. Hyperbilirubinemia: current guidelines and emerging therapies. Pediatr Emerg Care 2011; 27: 884–889. 21926893 10.1097/PEC.0b013e31822c9b4c

[bib36] 36Vetvicka V, Miler I, Sima P, Taborsky L, Fornusek L. The effect of bilirubin on the Fc receptor expression and phagocytic activity of mouse peritoneal macrophages. Folia Microbiol (Praha) 1985; 30: 373–380. 4029818 10.1007/BF02927593

[bib37] 37Granato A, Gores G, Vilei MT, Tolando R, Ferraresso C, Muraca M. Bilirubin inhibits bile acid induced apoptosis in rat hepatocytes. Gut 2003; 52: 1774–1778. 14633961 10.1136/gut.52.12.1774 PMC1773880

[bib38] 38Schmidt WN, Mathahs MM, Zhu Z. Heme and HO-1 Inhibition of HCV, HBV, and HIV. Front Pharmacol 2012; 3: 129–142. 23060790 10.3389/fphar.2012.00129 PMC3463857

[bib39] 39Jeong SH, Lee HS. Hepatitis A: clinical manifestations and management. Intervirology 2010; 53: 15–19. 20068336 10.1159/000252779

[bib40] 40Koff RS. Clinical manifestations and diagnosis of hepatitis A virus infection. Vaccine 1992; 10: S15–S17. 1335649 10.1016/0264-410x(92)90533-p

[bib41] 41Park SH, Rehermann B. Immune responses to HCV and other hepatitis viruses. Immunity 2014; 40: 13–24. 24439265 10.1016/j.immuni.2013.12.010 PMC4480226

[bib42] 42Zhou Y, Callendret B, Xu D, Brasky KM, Feng Z, Hensley LL et al. Dominance of the CD4(+) T helper cell response during acute resolving hepatitis A virus infection. J Exp Med 2012; 209: 1481–1492. 22753925 10.1084/jem.20111906 PMC3409494

[bib43] 43Manangeeswaran M, Jacques J, Tami C, Konduru K, Amharref N, Perrella O et al. Binding of hepatitis A virus to its cellular receptor 1 inhibits T-regulatory cell functions in humans. Gastroenterology 2012; 142: 1516–1525 e3. 22430395 10.1053/j.gastro.2012.02.039 PMC3367104

[bib44] 44Wu JF, Chiang BL, Chen HL, Lai HS, Chang MH, Ni YH. Impaired T-lymphocyte proliferation function in biliary atresia patients with chronic cholestatic jaundice after a Kasai operation. Pediatr Res 2006; 60: 602–606. 16966356 10.1203/01.PDR.0000242270.91973.ff

[bib45] 45Subhanova I, Muchova L, Lenicek M, Vreman HJ, Luksan O, Kubickova K et al. Expression of biliverdin reductase A in peripheral blood leukocytes is associated with treatment response in HCV-infected patients. PLoS One 2013; 8: e57555–e57561. 23536765 10.1371/journal.pone.0057555 PMC3594226

[bib46] 46Yamashita K, McDaid J, Ollinger R, Tsui TY, Berberat PO, Usheva A et al. Biliverdin, a natural product of heme catabolism, induces tolerance to cardiac allografts. FASEB J 2004; 18: 765–767. 14977878 10.1096/fj.03-0839fje

[bib47] 47Rocuts F, Zhang X, Yan J, Yue Y, Thomas M, Bach FH et al. Bilirubin promotes de novo generation of T regulatory cells. Cell Transplant 2010; 19: 443–451. 20021735 10.3727/096368909X484680

[bib48] 48Weinberger B, Archer FE, Kathiravan S, Hirsch DS, Kleinfeld AM, Vetrano AM et al. Effects of bilirubin on neutrophil responses in newborn infants. Neonatology 2013; 103: 105–111. 23182920 10.1159/000343097 PMC4834984

[bib49] 49Chatenoud L, Bach JF. Genetic control of hepatitis A severity and susceptibility to allergy. J Clin Invest 2011; 12: 848–850. 10.1172/JCI46418 PMC3049371 21339636

[bib50] 50Isogai H, Hirayama N. A possible molecular mechanism of immunomodulatory activity of bilirubin. Int J Med Chem 2013; 2013: 467383–467387. 25431670 10.1155/2013/467383 PMC4238221

[bib51] 51Blackard JT, Shata MT, Shire NJ, Sherman KE. Acute hepatitis C virus infection: a chronic problem. Hepatology 2008; 47: 321–331. 18161707 10.1002/hep.21902 PMC2277496

[bib52] 52Zhu Z, Mathahs MM, Schmidt WN. Restoration of type I interferon expression by heme and related tetrapyrroles through inhibition of NS3/4 A protease. J Infect Dis 2013; 208: 1653–1663. 23901085 10.1093/infdis/jit338 PMC6281408

[bib53] 53Zhu Z, Wilson AT, Luxon BA, Brown KE, Mathahs MM, Bandyopadhyay S et al. Biliverdin inhibits hepatitis C virus nonstructural 3/4A protease activity: mechanism for the antiviral effects of heme oxygenase? Hepatology 2010; 52: 1897–1905. 21105106 10.1002/hep.23921 PMC3058505

[bib54] 54Zhu Z, Wilson AT, Mathahs MM, Wen F, Brown KE, Luxon BA et al. Heme oxygenase-1 suppresses hepatitis C virus replication and increases resistance of hepatocytes to oxidant injury. Hepatology 2008; 48: 1430–1439. 18972446 10.1002/hep.22491 PMC2587102

[bib55] 55Barrett S, Kieran N, Ryan E, O'Keane J C, Crowe J. Intrahepatic hepatitis C viral RNA status of serum polymerase chain reaction-negative individuals with histological changes on liver biopsy. Hepatology 2001; 33: 1496–1502. 11391539 10.1053/jhep.2001.24372

[bib56] 56Cengiz M, Yilmaz G, Ozenirler S. The association between indirect bilirubin levels and liver fibrosis due to chronic hepatitis C virus infection. Pathol Res Pract 2014; 210: 488–493. 24842533 10.1016/j.prp.2014.04.001

